# Ischemic Stroke in a Young Adult Male During Finasteride Therapy

**DOI:** 10.7759/cureus.105537

**Published:** 2026-03-20

**Authors:** Ribal Houmani, Jad Houmani, Amira Hamou, Saad Aad, Ghassan Nabbout

**Affiliations:** 1 Department of Internal Medicine, Faculty of Medicine and Medical Sciences, University of Balamand, Beirut, LBN; 2 Faculty of Health and Sciences, University of Balamand, Beirut, LBN

**Keywords:** factor v leiden, finasteride, ischemic stroke, thrombophilia, young adult

## Abstract

Finasteride, a 5-alpha-reductase inhibitor widely prescribed for androgenetic alopecia, is generally considered safe, with most reported adverse effects being sexual or neuropsychiatric in nature. However, recent literature data have raised concerns regarding rare thrombotic complications. The mechanistic basis and clinical relevance of this association remain unclear, particularly in young individuals without traditional risk factors. We report a case of ischemic stroke with left internal carotid artery multilevel stenosis and capsulo-thalamic infarction in a 20-year-old previously healthy male taking finasteride 1 mg daily for androgenetic alopecia. Comprehensive evaluation revealed heterozygous Factor V Leiden mutation, MTHFR compound heterozygosity, PAI-1 4G allele carriage, and ACE D allele polymorphism. Cardioembolic evaluation was unremarkable, and autoimmune testing was negative. Finasteride was discontinued, and the patient was treated with antiplatelet therapy and rehabilitation, with partial neurological recovery. This case highlights ischemic stroke occurring in the setting of a likely intracranial steno-occlusive arteriopathy, with concurrent finasteride exposure as a possible compounding factor. While causality cannot be definitively established, clinicians should remain vigilant when prescribing finasteride to individuals with prothrombotic predispositions. Further observational studies are needed to clarify absolute risk.

## Introduction

Finasteride, a competitive 5-alpha-reductase inhibitor, is widely prescribed for androgenetic alopecia and benign prostatic hyperplasia, with an estimated 1-2 million men using it globally for hair loss prevention [1]. It effectively reduces dihydrotestosterone (DHT) levels by approximately 65-70% within 24 hours of administration [2]. While traditionally regarded as safe with predominantly sexual and neuropsychiatric adverse effects [1], emerging case reports and pharmacovigilance signals have raised concerns regarding potential thrombotic complications [3,4]. The proposed mechanism involves hormonal pathway disruption, whereby finasteride-mediated inhibition of 5-alpha-reductase leads to increased testosterone aromatization to estrogen, potentially creating a hypercoagulable state [3,5]. Cryptogenic stroke accounts for up to 25% of ischemic strokes in young adults despite comprehensive evaluation [6]. Identifying potential medication-related triggers is therefore essential for accurate risk stratification. We report a case of ischemic stroke in a 20-year-old male patient receiving finasteride therapy in the setting of inherited thrombophilia.

## Case presentation

A 20-year-old previously healthy male developed an acute left-sided headache lasting several minutes, followed by sudden generalized weakness and collapse. Symptoms resolved spontaneously within minutes and he resumed normal daily activities without seeking medical attention. The following morning, he awoke having persistent right-sided hemiparesis with predominant weakness of the right hand and dysarthria. Expecting spontaneous resolution as on the previous day, he delayed seeking medical care for approximately eight hours. He denied trauma, neck pain, seizures, or loss of consciousness. He was a non-smoker with no history of alcohol consumption or recreational drug use. He had no past medical or surgical history. Family history was negative for cerebrovascular disease or inherited thrombophilia. Vitals were stable upon admission. Neurological examination revealed right-sided hemiparesis, more pronounced in the upper extremity, with markedly reduced fine motor control of the right hand and dysarthria. Pupils were equal, round, and reactive to light and accommodation bilaterally, with no ptosis or anisocoria. Sensory examination was unremarkable. The patient initially denied taking any medications, but on further inquiry regarding non-prescription or cosmetic treatments, he reported daily use of finasteride 1 mg for six months for androgenetic alopecia. 

Routine laboratory investigations, including complete blood count, renal and hepatic function panels, fasting lipid profile, thyroid function tests, homocysteine, inflammatory markers, and glucose metabolism, were all within normal limits, confirming the absence of traditional cardiovascular or metabolic risk factors. Both transesophageal echocardiography (TEE) and CT chest were unremarkable. Comprehensive thrombophilia screening revealed multiple genetic variants: Factor V Leiden: heterozygous G1691A mutation, MTHFR gene: compound heterozygosity, PAI-1 gene: carrier of 4G allele, and ACE gene: carrier of D allele (Table 1). Additional coagulation studies, including protein C, protein S, and antithrombin III activity, were within normal limits.

**Table 1:** Comprehensive thrombophilia screening

Gene	Codon/Polymorphism	Result	Interpretation
Factor V	G1691A (Leiden)	One mutation detected	Heterozygous
Factor V	H1299R (R2)	No mutation detected	Normal
Factor II (Prothrombin)	G20210A	No mutation detected	Normal
Factor XIII	V34L	Two mutations detected	Mutant Homozygous
β-Fibrinogen	−455 G>A	No mutation detected	Normal
Plasminogen Activator Inhibitor-1 (PAI-1)	4G/5G	4G/5G	Heterozygous
Platelet Glycoprotein IIIa (GPIIIa, HPA-1)	a/b	Ia/Ia	Normal
Methylene Tetrahydrofolate Reductase (MTHFR)	C677T	One mutation detected	Heterozygous
Methylene Tetrahydrofolate Reductase (MTHFR)	A1298C	One mutation detected	Heterozygous
Angiotensin-Converting Enzyme (ACE) I/D	Deletion/insertion	Deletion/Insertion (D/D)	Heterozygous
Apolipoprotein B (Apo B)	R3500Q	No mutation detected	Normal
Apolipoprotein E (Apo E) – Codon 112: TGC (Cys)	–	Positive	- ApoE Genotype: E3/3
Apolipoprotein E (Apo E) – Codon 112: CGC (Arg)	–	Negative	
Apolipoprotein E (Apo E) – Codon 158: TGC (Cys)	–	Negative	
Apolipoprotein E (Apo E) – Codon 158: CGC (Arg)	–	Positive	

A comprehensive autoimmune panel was negative including ANA, lupus anticoagulant, antiphospholipid antibodies (anticardiolipin, β2-glycoprotein I), anti-SSA/SSB antibodies, ANCA (PR3 and MPO), centromere antibodies, Scl-70 antibodies, ribonuclear protein (U1-nRNP) antibodies, anti-Jo-1 IgG, and Smith (Sm) antibodies.

Radiographic findings

**Figure 1:**
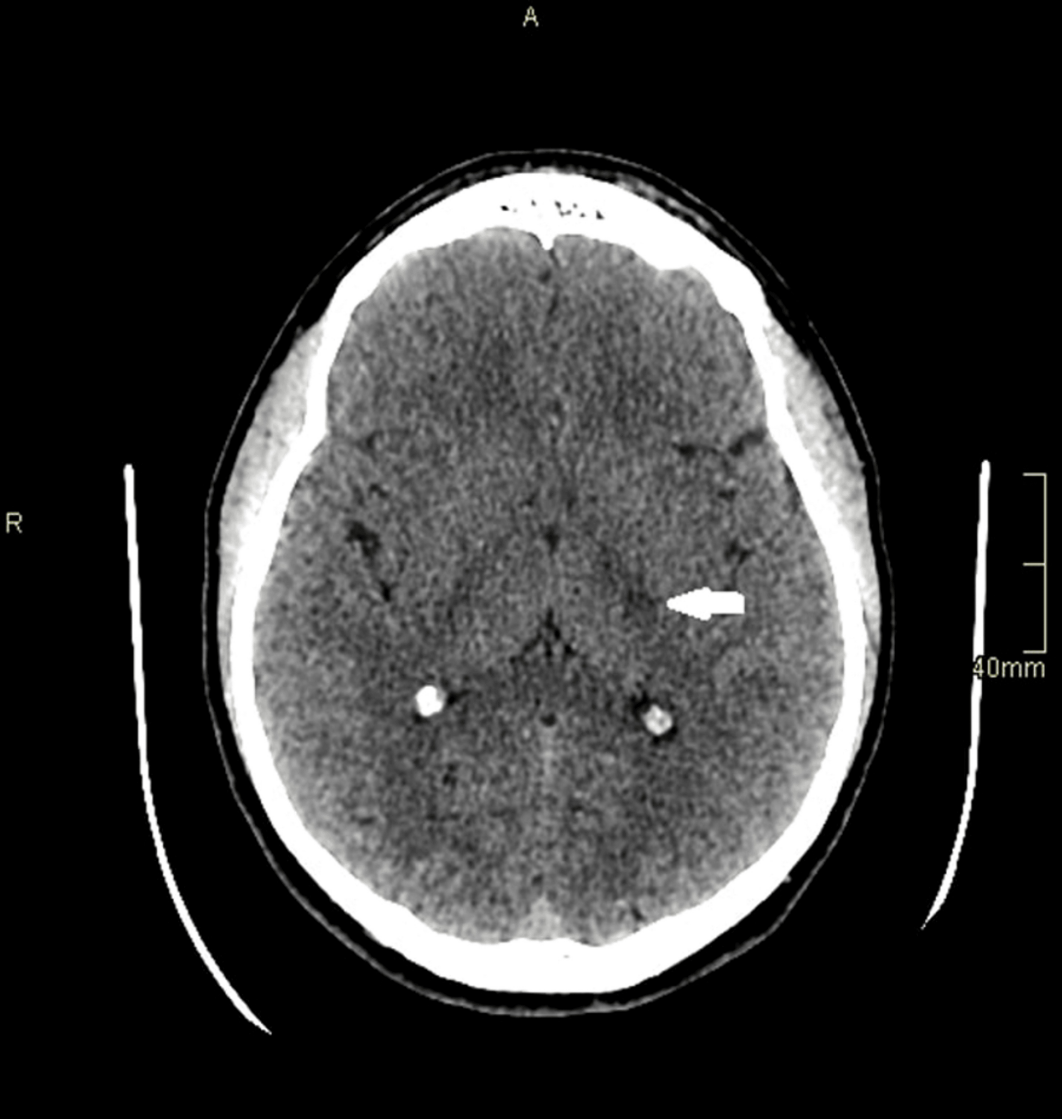
Non-contrast CT revealing left capsulo-thalamic hypodensity

A non-contrast CT scan showed left capsulo-thalamic hypodensity (Figure 1).

**Figure 2:**
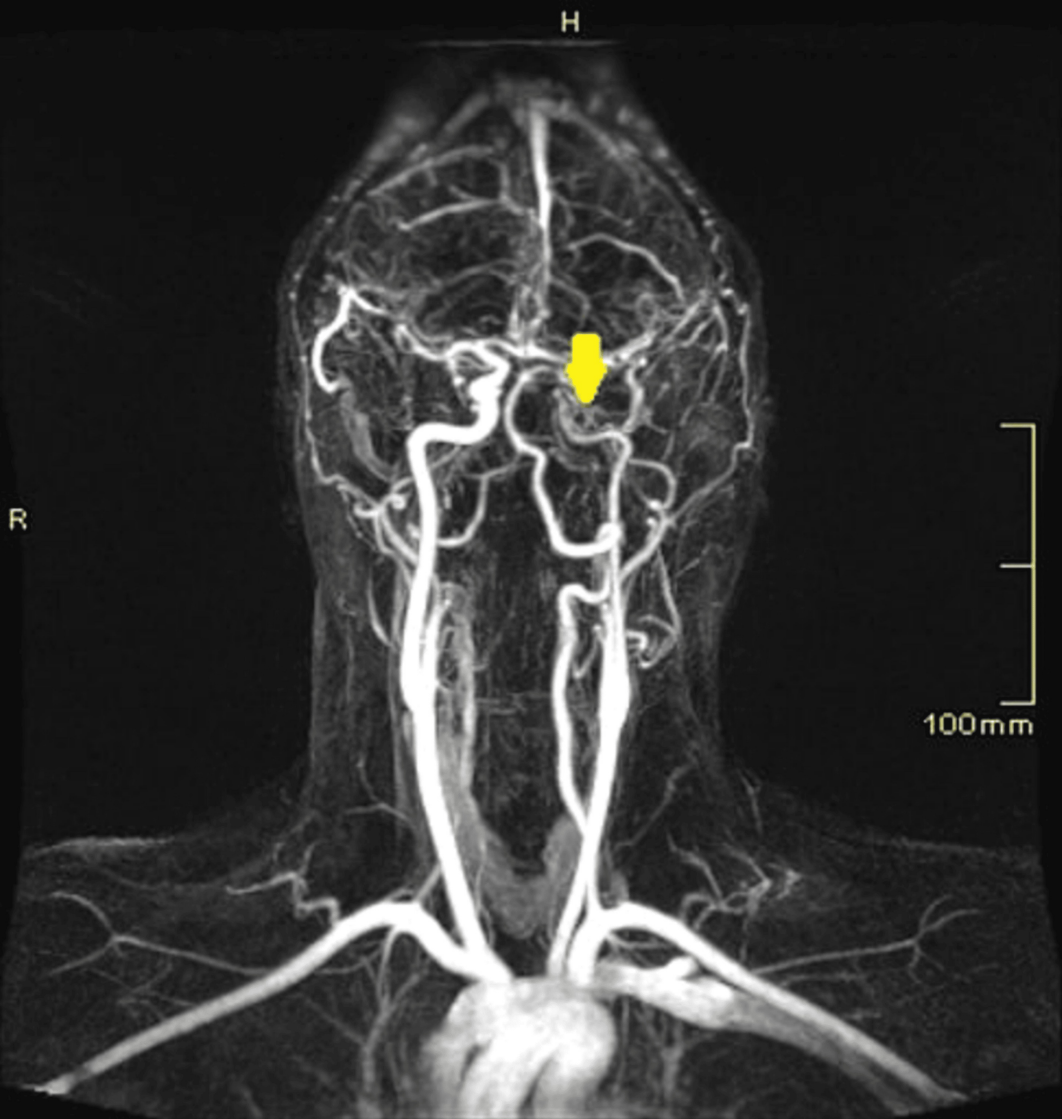
MRA demonstrating irregularities of the distal left ICA with multilevel significant stenosis ICA: Internal carotid artery; MRA: magnetic resonance angiography

**Figure 3:**
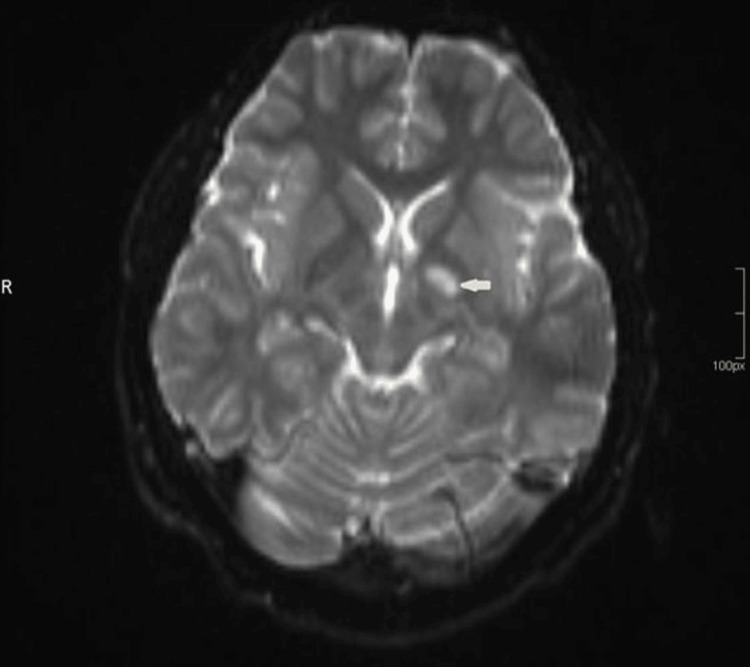
MRA showing left capsulo-thalamic restricted diffusion MRA: Magnetic resonance angiography

**Figure 4:**
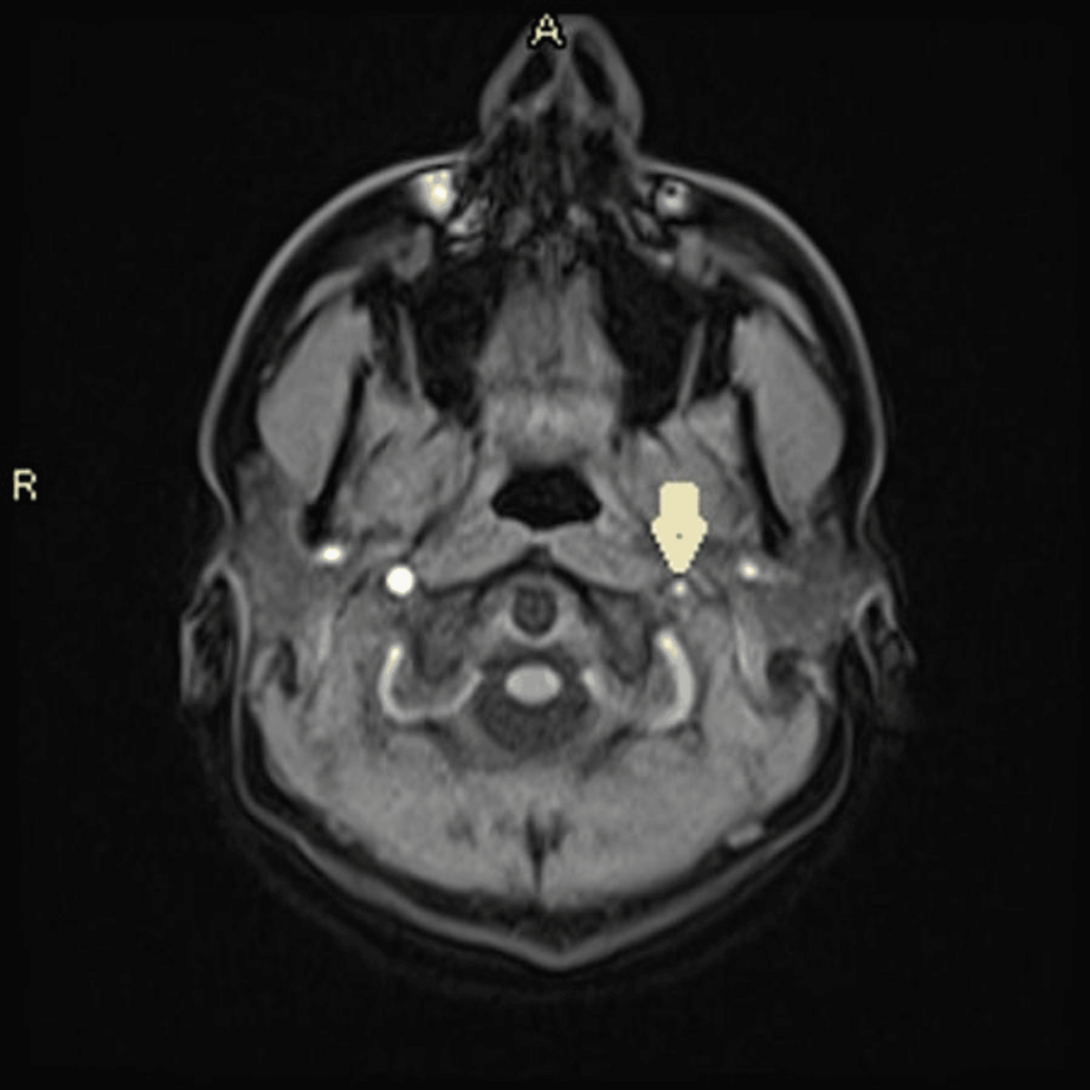
MRA showing narrowed left ICA ICA: Internal carotid artery; MRA: magnetic resonance angiography

Magnetic resonance angiography (MRA) revealed left internal carotid artery (ICA) irregularities with multilevel stenosis, acute capsulo-thalamic infarction, and restricted diffusion (Figures 2-4)

Conventional angiography was recommended to further characterize the intracranial steno‑occlusive changes; however, the patient declined the procedure because of concerns about its invasiveness.

Hospital course and treatment

Finasteride was promptly discontinued upon admission. The patient was initiated on antiplatelet therapy with aspirin 81 mg daily and received supportive care, including physical therapy. Over the hospital stay, neurological deficits improved, particularly in the right lower limb and speech, while moderate weakness persisted in the right hand. The patient was discharged on continued antiplatelet therapy with follow-up in neurology and rehabilitation services.

## Discussion

The Japan Pharmaceutical and Medical Devices Agency reported 14 thrombotic events between 2005 and 2014 among finasteride users, including four ischemic strokes and six myocardial infarctions [3]. Most reported patients were older and had additional vascular risk factors. A more recent case described a 21-year-old male with thalamic stroke during finasteride therapy, although a patent foramen ovale was identified, limiting causal attribution [4]. Thus, whether finasteride alone or in synergy with inherited thrombophilia can precipitate arterial thrombosis in otherwise healthy young adults remains uncertain.

Factor V Leiden is the most common inherited thrombophilia in Caucasian populations and is predominantly associated with venous thromboembolism, while isolated arterial ischemic stroke at a young age remains uncommon in the absence of additional prothrombotic triggers [7,8]. In contrast, heterozygosity for common MTHFR polymorphisms (C677T or A1298C) in the presence of normal homocysteine levels is not considered an independent risk factor for thrombosis [7]. The PAI-1 4G allele and ACE I/D polymorphism have been associated with mild increases in cardiovascular risk in population studies; however, their individual contribution to acute arterial thrombosis is limited and generally insufficient as a sole causative factor [7]. Not all rare hereditary or acquired thrombophilias were investigated, and some conditions therefore cannot be completely excluded. The autoimmune panel was negative, making autoimmune vasculitis or antiphospholipid syndrome unlikely. TEE was unremarkable, reducing the likelihood of cardiac-origin stroke. The absence of Horner's syndrome, neck pain, or trauma history further decreased the probability of spontaneous ICA dissection.

The proposed mechanism linking finasteride to thrombotic events centers on disrupted steroid hormone metabolism and its downstream effects on coagulation. Finasteride selectively inhibits type II and III 5-alpha-reductase isoenzymes, reducing DHT production by approximately 65-70% while simultaneously increasing circulating testosterone levels by 10-15% [2]. Testosterone undergoes peripheral aromatization to estradiol in adipose tissue and other organs, which can result in relative hyperestrogenemia, with estrogen levels rising by 10-15% during finasteride therapy [2]. The hypercoagulable effects of estrogen are well-established, involving increased synthesis of procoagulant factors (VII, X, and von Willebrand factor), enhanced platelet aggregability, and reduced anticoagulant protein activity [5]. While the magnitude of hormonal elevation with finasteride is typically modest in men, this hormonal shift may become clinically relevant in individuals with underlying thrombophilic predisposition.

The multilevel irregular stenosis and flow-related remodeling of the left ICA on MRA suggest an underlying intracranial steno-occlusive arteriopathy as the most likely primary etiology for the ischemic event. Conventional cerebral angiography remains the gold standard for diagnosing moyamoya angiopathy, particularly in unilateral or atypical cases [9]. In our patient, conventional angiography was advised but ultimately refused, and therefore early or unilateral moyamoya or other intracranial steno-occlusive disease cannot be excluded. Concurrent finasteride therapy may have acted as a compounding prothrombotic factor, precipitating an acute ischemic event on top of the pre-existing arteriopathy.

Routine universal thrombophilia screening prior to initiation of finasteride is not supported by current evidence. Individuals with established risk factors for thrombosis who are starting finasteride may reasonably be counseled about potential thrombotic symptoms, including sudden focal weakness, new speech disturbance, and severe or new headache, and advised to seek urgent medical attention should these occur. Topical finasteride formulations with reduced systemic absorption could be considered as a potentially safer alternative in carefully selected high‑risk individuals.

## Conclusions

This case highlights ischemic stroke occurring in the setting of a likely intracranial steno-occlusive arteriopathy, with concurrent finasteride exposure as a possible compounding factor. Given that conventional cerebral angiography was declined, the intracranial steno-occlusive lesion could not be fully characterized, and a primary intracranial arteriopathy such as moyamoya angiopathy cannot be excluded. Accordingly, the association between finasteride use and stroke in this patient should be regarded as hypothesis-generating rather than proof of causality. Larger observational studies are required to clarify the nature and magnitude of this association.

## References

[1] Estill MC, Ford A, Omeira R, Rodman M (2023). Finasteride and dutasteride for the treatment of male androgenetic alopecia: a review of efficacy and reproductive adverse effects. Georgetown Med Rev.

[2] Zito PM, Bistas KG, Patel P, Syed K (2025). Finasteride. StatPearls [Internet].

[3] Tsuji Y, Nakayama T, Bono K, Kitamura M, Imafuku I (2014). Two cases of stroke associated with the use of finasteride, an approved drug for male-pattern hair loss in Japan (Article in Japanese). Rinsho Shinkeigaku.

[4] Gaddameedi SR, Khan MA, Arty F, Bandari V, Vangala A, Panchal P, Shah SM (2024). An unusual case of thalamic stroke in a young adult with patent foramen ovale and finasteride use. Cureus.

[5] Coleman JR, Moore EE, Schmitt L (2023). Estradiol provokes hypercoagulability and affects fibrin biology: a mechanistic exploration of sex dimorphisms in coagulation. J Trauma Acute Care Surg.

[6] Ekker MS, Verhoeven JI, Schellekens MM (2023). Risk factors and causes of ischemic stroke in 1322 young adults. Stroke.

[7] Alnor AB, Gils C, Vinholt PJ (2024). Venous thromboembolism risk in adults with hereditary thrombophilia: a systematic review and meta-analysis. Ann Hematol.

[8] Albagoush SA, Koya S, Chakraborty RK, Schmidt AE (2025). Factor V Leiden mutation. StatPearls [Internet].

[9] Kuroda S, Fujimura M, Takahashi J (2022). Diagnostic criteria for Moyamoya disease - 2021 revised version. Neurol Med Chir (Tokyo).

